# Recent trends in chronic disease, impairment and disability among older adults in the United States

**DOI:** 10.1186/1471-2318-11-47

**Published:** 2011-08-18

**Authors:** William W Hung, Joseph S Ross, Kenneth S Boockvar, Albert L Siu

**Affiliations:** 1 Department of Geriatrics and Palliative Medicine, Mount Sinai School of Medicine, New York, NY, USA; 2 The Health Services Research and Development Research Enhancement Award Program and the Geriatrics Research, Education, and Clinical Center, James J. Peters Veterans Affairs Medical Center, Bronx, NY, USA; 3 Division of General Internal Medicine, Yale University School of Medicine and Center for Outcomes Research and Evaluation, Yale-New Haven Hospital, New Haven, CT, USA

**Keywords:** chronic disease, impairment, disability, prevalence trends

## Abstract

**Background:**

To examine concurrent prevalence trends of chronic disease, impairment and disability among older adults.

**Methods:**

We analyzed the 1998, 2004 and 2008 waves of the Health and Retirement Study, a nationally representative survey of older adults in the United States, and included 31,568 community dwelling adults aged 65 and over. Measurements include: prevalence of chronic diseases including hypertension, heart disease, stroke, diabetes, cancer, chronic lung disease and arthritis; prevalence of impairments, including impairments of cognition, vision, hearing, mobility, and urinary incontinence; prevalence of disability, including activities of daily living (ADLs) and instrumental activities of daily living (IADLs).

**Results:**

The proportion of older adults reporting no chronic disease decreased from 13.1% (95% Confidence Interval [CI], 12.4%-13.8%) in 1998 to 7.8% (95% CI, 7.2%-8.4%) in 2008, whereas the proportion reporting 1 or more chronic diseases increased from 86.9% (95% CI, 86.2%-89.6%) in 1998 to 92.2% (95% CI, 91.6%-92.8%) in 2008. In addition, the proportion reporting 4 or more diseases increased from 11.7% (95% CI, 11.0%-12.4%) in 1998 to 17.4% (95% CI, 16.6%-18.2%) in 2008. The proportion of older adults reporting no impairments was 47.3% (95% CI, 46.3%-48.4%) in 1998 and 44.4% (95% CI, 43.3%-45.5%) in 2008, whereas the proportion of respondents reporting 3 or more was 7.2% (95% CI, 6.7%-7.7%) in 1998 and 7.3% (95% CI, 6.8%-7.9%) in 2008. The proportion of older adults reporting any ADL or IADL disability was 26.3% (95% CI, 25.4%-27.2%) in 1998 and 25.4% (95% CI, 24.5%-26.3%) in 2008.

**Conclusions:**

Multiple chronic disease is increasingly prevalent among older U.S. adults, whereas the prevalence of impairment and disability, while substantial, remain stable.

## Background

Disability, such as the inability to dress, bathe or manage one's medications, is prevalent and costly among older adults in the United States. The development of such disability among older adults is often complex and multifactorial [1-3]. Many have postulated that co-morbid chronic diseases are significant risk factors for developing disabilities [1]. Over the past 20 years, there has been a rise in chronic disease prevalence [4,5], and the majority of the older population over age 65 now suffers from multiple chronic diseases [4,6,7]. However, recent literature on the patterns of chronic diseases and disability found that despite the increase in the prevalence of chronic diseases, disability prevalence has declined [8-14], although not uniformly across groups of different age and sex [15-17]. Populations in other developed countries, such as the Netherlands, have also observed increases in chronic disease prevalence but stable or declines in disability rates [18,19]. However, trends in disability rates among developed countries are not uniformly stable or in decline, as Belgium, Japan and Sweden reported increases in disability [20].

Considering the pathway to disability, impairments have been considered an intermediary between chronic diseases and disability [1,2]. For example, a person with chronic arthritis may develop mobility impairment; or a person with diabetes mellitus may develop diabetic retinopathy which impairs vision. In turn, the mobility or visually impaired person may become unable to carry out tasks which are essential for independent living. Prior examinations of individual impairments--such as impairments in vision, hearing and cognition [21-23] -- have suggested that they may have become less prevalent over time, but other studies have contradicted these findings, demonstrating that there were no significant changes [24,25]. The patterns of impairments, which disproportionately affect older adults, and multiple impairments over time are not well known. Given the dynamics between chronic disease, impairment and disability, and that significant changes have occurred recently in terms of socio-demographic makeup and health care among older adults, an up to date examination of the trends of impairments and disability among older adults may improve our current understanding of health care needs for this population.

Therefore our primary objective is to examine the trends of impairments among older adults in the context of increasing trends of chronic diseases, and opposing trends of disability, in order to better describe the health care needs of older adults. A second objective is to use the most recent data to examine the continuing detailed trends of chronic diseases and disability stratified by age and sex. The Health and Retirement Study (HRS) [26], which is a nationally representative survey of older adults in the US, surveys adults on multiple impairments and disability, and thus, provides an excellent opportunity to examine these concurrent trends among older adults.

## Methods

The Health and Retirement Study (HRS) [26] is a national longitudinal survey of U.S. adults over age 50 sponsored by the National Institute on Aging and conducted by the Institute for Social Research at the University of Michigan. The study was designed to investigate the experience of aging among older adults as they advance from work to retirement with particular emphasis on health insurance, savings, and trajectories of economic and physical well-being. The initial wave of the HRS conducted in 1992 comprised the core sample of the HRS and included 12,652 community-dwelling adults between the ages of 51 and 61 or their spouses, regardless of age, collected via in-home interviews. The sample was combined in 1998 with the Asset and Health Dynamics among the Oldest-Old Study (AHEAD) [27,28], which is a survey of a nationally representative sample of persons who were born in 1923 or before. Subsequent waves were supplemented with new birth cohorts every 6 years so as to be representative of the entire US household population aged 50 and above. A full description of the procedures used in the HRS surveys has been published previously [26]. The survey data are publicly available and do not contain any unique identifiers. Data from three waves of interviews were collated for the present analysis: 1998, 2004 and 2008. We only included older adults who were at least 65 years of age at the times of the interview.

### Chronic Disease Definition

In each wave, respondents were asked to self-report whether they had ever been told by their physician that they had any of several chronic diseases. Proxy respondents were surveyed if the respondents were not able to participate in the survey. Chronic diseases surveyed in all three waves of the HRS studied included hypertension, heart conditions (which included coronary heart diseases and heart failure), chronic lung disease (such as chronic bronchitis or emphysema, but not asthma), diabetes, stroke (which also included transient ischemic attack), cancer (or a malignant tumor of any kind except skin cancer), and arthritis. We then categorized respondents by whether they self-reported 0, 1, 2, 3 and 4 or more chronic diseases.

### Impairment Definition

In each wave, respondents were asked to self-report whether they had any of several impairments. We used a similar approach as Cigolle et al [29] in defining impairments and included several impairments self-reported by respondents: (1) vision (fair or poor eyesight or blindness despite the use of glasses or corrective lenses as usual); (2) hearing (fair or poor hearing despite the use of a hearing aid as usual); and (3) cognition. Cognition was measured in each wave using a standardized test on a 35 point scale [30], with higher scores indicating better cognitive function. This scale was modified from the Telephone Interview for Cognitive Status and contained similar items to the Mini Mental Status Examination [31], and has been validated as a cognitive screening instrument. We defined cognitive impairment as a score at or below 8, which has been previously used as cutoff score for severe cognitive impairment because it was consistent with other estimates of the prevalence of dementia [32]. In addition, for respondents who were unable to complete the survey and had proxy respondents, proxy reports of fair or poor memory were considered to be indicative of cognitive impairment of the respondent, as has been done in prior research [33].

We further included two other impairments commonly seen in older adults: urinary incontinence and mobility impairment. Respondents with loss of control of their urination in the past 12 months were characterized to have urinary incontinence. Prior national survey data such as the National Health and Nutrition Examination Survey (NHANES) also use similar definition for estimating the prevalence of urinary incontinence [34]. Respondents who reported difficulty in ambulating across the room were characterized as having mobility impairment. This measure has previously been used in other large scale studies such as the Women's Health and Aging Study as a marker for severe mobility difficulty [35]. In total, we characterized 5 conditions as indicative of impairment (visual, hearing, cognitive, mobility impairment and urinary incontinence). We then categorized respondents by whether they self-reported 0, 1, 2, and 3 or more impairments.

### Disability Definition

In each wave, respondents were asked to self-report whether they had any difficulty with any of several activities of daily living (ADL) and instrumental activities of daily living (IADL). The following ADLs were surveyed in each of the three survey waves: bathing, dressing, eating, toileting and transferring; and the following IADLs were surveyed: using the telephone, managing money, managing medications, grocery shopping and preparation of meals. We characterized respondents as having disability in a task if they reported difficulty, or received help for the task, or could not perform the task secondary to health reasons. We then categorized respondents by whether they self-reported any disability in ADL tasks, in IADL tasks, and in either ADL or IADL tasks.

### Other Variables

Several other socio-demographic variables were included in our analyses for the purposes of describing changes in population characteristics: age, sex, race, marital status and education. We categorized race into categories of white and non-white, marital status into categories of married and not married, and education into categories of under 8^th ^grade education, 8^th ^to 11^th ^grade education, high school education, and beyond high school education. We also included body mass index and current smoking status as indicators of body size and health behaviors.

### Statistical Analysis

Respondent characteristics were summarized for each wave of the HRS: 1998, 2004, and 2008. Next we described the prevalence of each chronic disease, impairment and disability in each wave, and summarized the prevalence of multiple chronic diseases and multiple impairments in each wave. We examined the concurrent trends of multiple chronic diseases, impairments and disability across all three survey years. We then stratified each sample into age groups of 65-69, 70-74, 75-79, 80-84 and 85 and above and by sex, and examined the proportions of respondents with different numbers of chronic diseases, impairments, and disability. The purpose of stratifying in 5 year groups is to (1) examine whether there are age dependent trends and (2) to clarify that the changes in chronic disease, impairment, and disability prevalence were not due to the accumulation of diseases in the same cohort over time. We performed sensitivity analysis by removing each individual chronic disease and impairment from the total number of conditions to test if observed trends were due to one condition only. We used weighted chi square test with Wald distribution to test group differences in proportions and one way ANOVA F-test for continuous variables. We used a p-value threshold of 0.05 for statistical significance. We applied sampling weights according to the methodology described in the HRS manual for the application of sampling weights for respondent level characteristics [36]. The respondent level weight is non-zero for living non-institutionalized respondents in the appropriate years. It is scaled so as to yield weight sums which correspond to the number of individuals in the U.S. population as measured by the March Current Population Survey (CPS) for the year of data collection. All analyses were performed using Stata version 8.0 (Stata Corp LP, College Station, TX). Because the HRS is a publicly available anonymous data source, our study was exempted from review by the Mount Sinai Institutional Review Board.

## Results

A total of 10,390, 10,621 and 10,557 respondents were included in the HRS waves in 1998, 2004 and 2008, respectively. Among all three survey waves, the average age was 74.6, 57.3% were female, 89.1% were white, and 55.1% were married (Table 1) and there were no clinically significant differences in the age, sex, race, and marital status across all three survey years. However, education levels and average BMI rose in subsequent survey years, from 33.1% to 40.3% completing an education above high school and from 26.0 to 27.5 kg/m^2 ^respectively.

**Table 1:** Descriptive statistics of adults aged 65 and over in the Health and Retirement Study (HRS) in 1998, 2004 and 2008

Demographic characteristics	Overall	1998 (n = 10390)	2004 (n = 10621)	2008 (n = 10557)	p-value
Mean age, y (95% CI)	74.6 (74.5, 74.7)	74.4 (74.3, 74.5)	74.9 (74.7, 75.0)	74.5 (74.3, 74.6)	< 0.001

Female sex, % (95%CI)	57.3 (56.7, 57.9)	58.1 (57.0, 59.1)	57.1 (56.0, 58.1)	56.9 (55.9, 58.0)	0.26

Race					0.40
White, % (95% CI)	89.5 (89.2, 89.8)	89.4 (88.8, 89.9)	89.8 (89.3,90.4)	89.4 (88.8,90.0)	
Black, % (95% CI)	8.3 (8.0, 8.6)	8.5 (8.0, 9.0)	8.1 (7.6, 8.6)	8.3 (7.9, 8.8)	
Other, % (95% CI)	2.2 (2.0, 2.4)	2.1 (1.8, 2.4)	2.1 (1.8, 2.4)	2.3 (2.0, 2.6)	

Married, % (95% CI)	55.1 (54.4, 55.7)	55.3 (54.3, 56.4)	55.9 (54.9, 56.9)	54.1 (53.0, 55.2)	0.05

Education, % (95% CI)					< 0.001
Under 8^th ^grade	7.5 (7.2, 7.8)	9.6 (9.1, 10.2)	7.1 (6.6, 7.6)	6.0 (5.6, 7.5)	
8-11 grade	20.6 (20.1, 21.1)	24.2 (23.4, 25.1)	20.2 (19.4, 21.1)	18.0 (20.1, 21.1)	
High school	34.9 (34.4, 35.5)	33.1 (32.1, 34.1)	35.8 (34.8, 36.8)	35.7 (34.4, 35.5)	
Above high school	37.0 (36.4, 37.6)	33.1 (32.1, 34.1)	36.9 (35.9, 37.9)	40.3 (36.4, 37.6)	

Current Smoker, % (95% CI)	10.1 (9.7, 10.4)	10.9 (10.3, 11.6)	9.3 (8.8, 10.0)	10.1 (9.4, 10.8)	0.002

Body Mass Index, (95% CI)	26.7 (26.6, 26.8)	26.0 (25.9, 26.1)	26.6 (26.4, 26.7)	27.5 (27.3, 27.6)	< 0.001

**Chronic diseases, %**					
**(95%CI)**					
Hypertension	59.7 (59.2, 60.3)	52.5 (51.5, 53.5)	60.5 (59.5, 61.5)	65.0 (63.9, 66.0)	< 0.001
Heart conditions	31.4 (30.8, 32.0)	30.7 (29.7, 31.6)	31.9 (30.9, 32.9)	31.6 (30.6, 32.5)	0.20
Stroke	9.3 (9.0, 9.7)	9.8 (9.2, 10.4)	8.9 (8.4, 9.6)	9.2 (8.6, 9.8)	0.14
Diabetes	19.3 (18.8, 19.8)	15.2 (14.5, 15.9)	19.3 (18.4, 20.1)	22.7 (21.8, 23.6)	< 0.001
Cancer	17.4 (16.9, 17.9)	14.6 (13.9, 15.3)	18.1 (17.3, 18.9)	19.1 (18.2, 19.9)	< 0.001
Chronic lung disease	11.6 (11.2, 12.0)	10.8 (10.1, 11.4)	11.6 (10.9, 12.3)	12.3 (11.6, 13.0)	0.01
Arthritis	65.5 (64.9, 66.1)	59.1 (58.1, 60.1)	67.7 (66.7, 68.6)	68.8 (67.8, 69.8)	< 0.001

**Impairments, %**					
**(95% CI)**					
Severe Cognitive Impairment	4.3 (4.1, 4.6)	4.9 (4.5, 5.3)	4.2 (3.8, 4.5)	4.0 (3.6, 4.4)	0.01
Visual Impairment	23.7 (23.2, 24.2)	25.4 (24.5, 26.3)	23.6 (22.7, 24.5)	22.4 (21.6, 23.3)	< 0.001
Hearing Impairment	25.8 (25.3, 26.4)	25.4 (24.5, 26.3)	27.2 (26.2, 28.1)	25.0 (24.1, 25.9)	0.003
Urinary incontinence	23.8 (23.3, 24.4)	19.8 (19.0, 20.6)	23.6 (22.7, 24.4)	27.5 (26.5, 28.4)	< 0.001
Mobility Impairment	8.2 (7.9, 8.6)	8.3 (7.8, 8.9)	7.9 (7.3, 8.4)	8.5 (7.9, 9.1)	0.27

**Disability, % (95%**					
**CI)**					
**ADL dependence** Bath	8.6 (8.3, 9.0)	9.8 (9.2, 10.4)	8.2 (7.6, 8.8)	8.1 (7.5, 8.6)	0.001
Bed transfer	6.0 (5.7, 6.3)	6.9 (6.4, 7.4)	5.3 (4.9, 5.8)	5.9 (5.4, 6.4)	0.001
Dress Eating	10.9 (10.5, 11.3) 3.8 (3.6, 4.1)	11.5 (10.8, 12.1) 4.1 (3.7, 4.5)	10.2 (9.5, 10.8) 3.7 (3.3, 4.1)	11.0 (10.4,11.7) 3.8 (3.4, 4.2)	0.01 0.28
Toileting	6.3 (6.1, 6.6)	6.9 (6.4, 7.5)	5.8 (5.3, 6.3)	6.4 (5.8, 6.9)	0.01
**IADL dependence**					
Meal preparation	7.6 (7.2, 7.9)	7.8 (7.2, 8.3)	7.5 (7.0, 8.1)	7.4 (6.9, 8.0)	0.66
Medication management	4.0 (3.8, 4.2)	3.9 (3.5, 4.2)	3.9 (3.5, 4.3)	4.2 (3.8, 4.6)	0.53
Money management	7.1 (6.8, 7.5)	7.4 (6.9, 7.9)	7.0 (6.5, 7.5)	7.1 (6.5, 7.6)	0.56
Shopping	11.1 (10.7, 11.4)	11.7 (11.0, 12.3)	11.2 (10.6, 11.9)	10.4 (9.8, 11.0)	0.02
Telephone use	5.6 (5.3, 5.9)	5.9 (5.5, 6.4)	5.6 (5.1, 6.1)	5.6 (4.8, 5.7)	0.15

Abbreviations: CI, confidence interval; ADL, activities of daily living; IADL, instrumental activities of daily living.

Note: All estimates used sampling weights to account for survey design (with 95% confidence intervals in parentheses); p- values were derived from the Wald chi-square tests for categorical variables and ANOVA F-test for continuous variables for association between the proportion or characteristics of respondents and year of survey.

### Chronic Disease Trends

**Figure 1:**
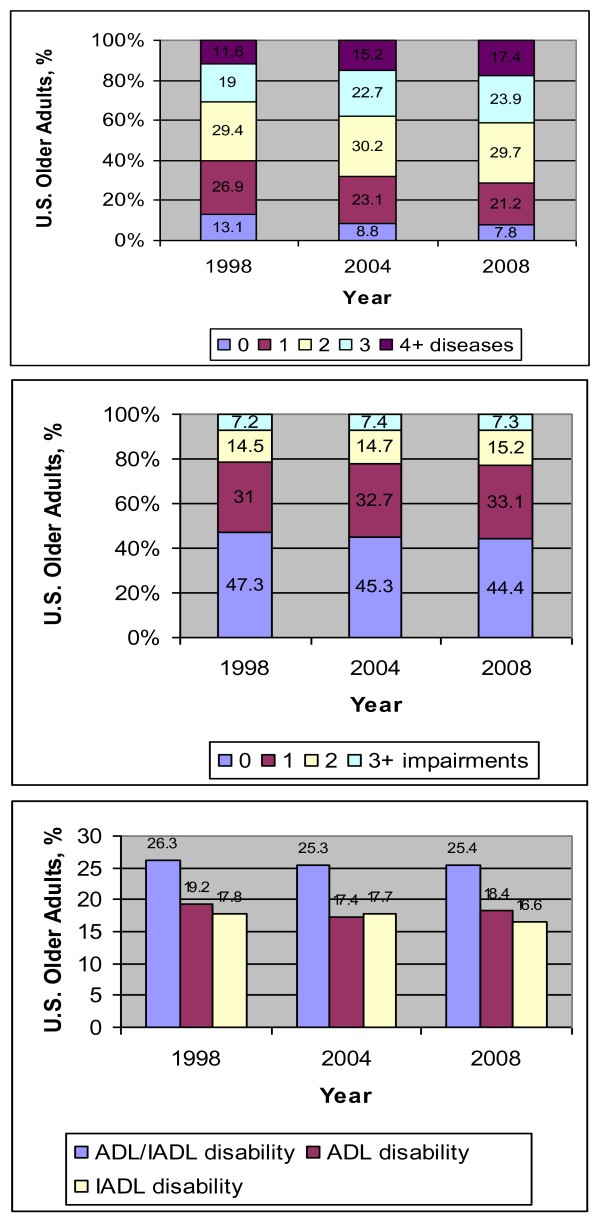
**Percentage of older adults aged 65 and over who have 0, 1, 2, 3, or 4 or more chronic diseases (A), 0, 1, 2, or 3 or more impairments (B) and disability in activities in daily living, instrumental activities of daily living or either (C) in the Health and Retirement Study in 1998, 2004 and 2008**. All estimates used sampling weights to account for survey design.

Among the chronic diseases examined, nearly all diseases, including hypertension, diabetes, cancer, chronic lung disease and arthritis showed an increasing trend throughout the study period (Table 1). On the other hand, the prevalence of heart conditions increased initially from 1998 to 2004 and subsequently remained stable as of 2008. The proportion of respondents reporting varying number of chronic diseases changed over the study period (Table 2 and Figure 1A in the online supplement; p value < 0.001). The proportion reporting no chronic disease decreased from 13.1% in 1998 to 8.8% in 2004 to 7.8% in 2008, as did the proportion reporting 1 chronic disease, from 26.9% in 1998 to 23.1% in 2004 to 21.2% in 2008. The proportion reporting 1 or more chronic diseases increased from 86.9% in 1998 to 91.2% in 2004 to 92.2% in 2008. In contrast, the proportion reporting 3 chronic diseases increased from 19.1% in 1998 to 22.7% in 2004 to 23.9% in 2008, as did the proportion reporting 4 or more chronic diseases, from 11.6% in 1998 to 15.2% in 2004 to 17.4% in 2008.

**Table 2:** Percentage of respondents with 0, 1, 2, 3, or 4 or more chronic diseases by survey year, overall and stratified by age and by sex

	Number of chronic diseases, % (95% Confidence Interval)				
	**0**	**1**	**2**	**3**	**4+**

Overall*					

1998, n = 10390	13.1 (12.4, 13.8)	26.9 (26.0, 27.8)	29.4 (28.5, 30.3)	19.1 (18.3, 19.9)	11.6 (10.9, 12.2)
2004, n = 10621	8.8 (8.2, 9.4)	23.1 (22.3, 24.0)	30.2 (29.2, 31.1)	22.7 (21.8, 23.6)	15.2 (14.5, 16.0)
2008, n = 10557	7.8 (7.2, 8.4)	21.2 (20.4, 22.1)	29.7 (28.8, 30.7)	23.9 (23.0, 24.8)	17.4 (16.6, 18.2)

**Respondent Age**					

65-69 years*					

1998, n = 3018	17.0 (15.5, 18.5)	30.8 (29.1, 32.6)	27.0 (25.3, 28.8)	16.1 (14.7, 17.5)	9.1 (8.1, 10.3)
2004, n = 3392	12.2 (11.0, 13.5)	26.9 (25.3, 28.6)	29.4 (27.8, 31.1)	19.8 (18.3, 21.3)	11.7 (10.6, 12.9)
2008, n = 3128	11.7 (10.3, 13.2)	25.0 (23.2, 26.9)	30.3 (28.4, 32.3)	20.4 (18.8, 22.1)	12.7 (11.4, 14.2)

70-74 years*					

1998, n = 2720	14.8 (13.4, 16.3)	27.7 (26.0, 29.5)	30.1 (28.3, 31.9)	17.6 (16.2, 19.2)	9.8 (8.7, 11.0)
2004, n = 2656	10.4 (9.2, 11.8)	24.3 (22.6, 26.2)	29.5 (27.6, 31.4)	21.5 (19.9, 23.3)	14.2 (12.8, 15.8)
2008, n = 2908	7.5 (6.5, 8.6)	21.7 (20.1, 23.5)	30.8 (28.9, 32.7)	23.2 (21.5, 25.0)	16.8 (15.4, 18.4)

75-79 years*					

1998, n = 2284	11.2 (9.9, 12.7)	24.8 (22.9, 26.7)	30.0 (28.0, 32.1)	20.3 (18.6, 22.2)	13.7 (12.2, 15.3)
2004, n = 2031	6.4 (5.3, 7.6)	20.9 (19.0, 22.9)	32.0 (29.8, 34.2)	24.1 (22.2, 26.2)	16.6 (14.9, 18.5)
2008, n = 2180	5.7 (4.7, 6.9)	19.3 (17.5, 21.3)	28.5 (26.4, 30.6)	26.6 (24.6, 28.7)	19.9 (18.1, 21.9)

80-84 years*					

1998, n = 1465	8.3 (6.9, 10.1)	23.7 (21.4, 26.2)	30.1 (27.6, 32.8)	22.6 (20.3, 25.0)	15.3 (13.3, 17.5)
2004, n = 1609	5.9 (4.8, 7.3)	19.1 (17.0, 21.4)	30.3 (27.9, 32.9)	25.2 (22.9, 27.7)	19.5 (17.4, 21.7)
2008, n = 1517	4.6 (3.6, 5.9)	16.8 (14.9, 19.0)	30.6 (28.1, 33.2)	28.1 (25.7, 30.7)	19.8 (17.7, 22.1)

≥ 85 years*					

1998, n = 1306	7.7 (6.1, 9.5)	22.2 (19.6, 24.9)	32.0 (29.1, 35.0)	24.4 (21.8, 27.3)	13.8 (11.8, 16.1)
2004, n = 1425 2008, n = 1588	5.0 (3.8, 6.6) 4.5 (4.6, 6.2)	20.7 (18.3, 23.3) 17.7 (18.2, 21.0)	30.0 (27.3, 32.9) 27.4 (27.8, 31.1)	26.4 (23.7, 29.2) 26.3 (24.1, 27.2)	17.9 (15.6, 20.5) 24.1 (18.7, 21.6)

**Respondent Sex**					

Male*					

1998, n = 4463	13.6 (12.6, 14.8)	27.2 (25.8, 28.6)	28.7 (27.3, 30.1)	19.2 (17.9, 20.4)	11.3 (10.4, 12.4)
2004, n = 4595	9.8 (8.9, 10.7)	22.7 (21.4, 24.1)	29.2 (27.8, 30.7)	23.0 (21.7, 24.4)	15.4 (14.3, 16.5)
2008, n = 4441	8.9 (7.9, 9.9)	21.1 (19.8, 22.6)	28.4 (26.9, 30.0)	24.1 (22.7, 25.5)	17.5 (16.3, 18.7)

Female*					

1998, n = 5927	12.8 (11.9, 13.7)	26.7 (25.5, 27.9)	29.9 (28.7, 31.2)	19.0 (17.9, 20.1)	11.7 (10.9, 12.6)
2004, n = 6026	8.1 (7.3, 8.8)	23.5 (22.3, 24.7)	30.9 (29.7, 32.2)	22.4 (21.3, 23.6)	15.1 (14.2, 16.1)
2008, n = 6116	6.9 (6.2, 7.7)	21.3 (20.2, 22.5)	30.7 (29.5, 32.0)	23.8 (22.6, 25.0)	17.3 (16.2, 18.4)

Values are weighted percentages, with 95% confidence intervals in parentheses, derived by using the HRS respondent population weights of each year to adjust for the sampling design of survey.

* P value < 0.001, derived from the Wald chi-square tests which were performed to examine group differences in proportions of characteristics across years of survey. P-values refer to whether the proportions or characteristics were significantly different among groups between years.

### Impairment Trends

Among impairments, the prevalence of hearing and mobility impairment remained stable, whereas the prevalence of visual and cognitive impairment declined slightly (Table 1). Urinary incontinence, however, increased in prevalence. The proportion of respondents reporting varying number of impairments changed over the study period (Table 3 and Figure 1B in the online supplement; p = 0.001). The proportion of respondents reporting no impairments declined slightly from 47.3% in 1998 to 45.3% in 2004 and 44.4% in 2008, whereas the proportion reporting 1 impairment increased slightly, from 31.0% in 1998 to 32.7% in 2004 to 33.1% in 2008. The proportion of respondents reporting 2 or 3 or more impairments remained stable.

**Table 3:** Percentage of respondents with 0, 1, 2, or 3 or more impairments by survey year, overall, and stratified by age and by sex

	Number of Impairments, % (95% Confidence Interval)			
	**0**	**1**	**2**	**3+**

Overall*				

1998, n = 10390	47.3 (46.3, 48.4)	31.0 (30.0, 31.9)	14.5 (13.8, 15.3)	7.2 (6.7, 7.7)
2004, n = 10621	45.3 (44.2, 46.3)	32.7 (31.6, 33.7)	14.7 (13.9, 15.4)	7.4 (6.9, 8.0)
2008, n = 10557	44.4 (43.3, 45.5)	33.1 (32.1, 34.1)	15.2 (14.5, 16.0)	7.3 (6.8, 7.9)

**Respondent Age**				

**65-69 years†**				

1998, n = 3018	58.8 (56.9, 60.7)	28.6 (26.9, 30.4)	9.1 (8.0, 10.2)	3.6 (2.9, 4.3)
2004, n = 3392	55.7 (53.8, 57.5)	30.1 (28.4, 31.8)	10.2 (9.1, 11.4)	4.1 (3.4, 4.9)
2008, n = 3128	53.0 (50.9, 55.1)	32.2 (30.3, 34.2)	11.2 (9.9, 12.6)	3.6 (2.9, 4.5)

**70-74 years**				

1998, n = 2720	51.4 (49.3, 53.3)	31.5 (29.6, 33.3)	12.4 (11.1, 13.7)	4.7 (4.1, 5.8)
2004, n = 2656	49.2 (47.1, 51.2)	32.9 (30.9, 34.9)	12.5 (11.2, 14.0)	5.4 (4.6, 6.5)
2008, n = 2908	49.1 (47.1, 51.2)	32.7 (30.9, 34.7)	13.5 (12.1, 14.9)	4.7 (3.9, 5.6)

**75-79 years**				

1998, n = 2284	44.5 (42.2, 46.7)	33.4 (31.3, 35.5)	15.2 (13.6, 16.8)	7.1 (6.0, 8.3)
2004, n = 2031	43.2 (40.9, 45.6)	35 (32.7, 37.2)	15.6 (14.0, 17.4)	6.3 (5.3, 7.5)
2008, n = 2180	41.2 (38.9, 43.5)	36.5 (34.3, 38.8)	15.3 (13.7, 17.1)	7 (5.8, 8.3)

**80-84 years**				

1998, n = 1465	35.1 (32.4, 37.9)	32.8 (30.2, 35.6)	21.5 (19.2, 23.9)	10.6 (9.0, 12.5)
2004, n = 1609	38.1 (35.5, 40.8)	35.2 (32.7, 37.9)	17.1 (15.1, 19.2)	9.6 (8.2, 11.3)
2008, n = 1517	36.3 (33.7, 39.1)	34.7 (32.1, 37.4)	18.7 (16.7, 20.9)	10.2 (8.7, 12.0)

**≥ 85 years**				

1998, n = 1306	24.1 (21.5, 27.0)	28.4 (25.6, 31.3)	26.4 (23.8, 29.3)	21 (18.6, 23.8)
2004, n = 1425	23.6 (21.0, 26.3)	30.9 (28.1, 33.8)	25.8 (23.2, 28.6)	19.8 (17.4, 22.4)
2008, n = 1588	25.5 (23.0, 28.2)	29.5 (26.9, 32.2)	25.5 (23.0, 28.2)	19.5 (17.3, 21.9)

**Respondent Sex**				

**Male**				

1998, n = 4463	47.5 (45.9,49.1)	32.2 (30.7, 33.7)	14.2 (13.1,15.3)	6.2 (5.5, 6.9)
2004, n = 4595	47.5 (45.9, 49.0)	31.9 (30.4, 33.4)	14.4 (13.3, 15.5)	6.3 (5.6, 7.1)
2008, n = 4441	47.8 (46.1, 49.5)	32.3 (30.7, 33.8)	14.5 (13.3, 15.7)	5.5 (4.8, 6.2)

Female*				

1998, n = 5927	47.2 (46.9, 48.6)	30.1 (28.9, 31.4)	14.8 (13.8, 15.8)	7.9 (7.2, 8.7)
2004, n = 6026	43.6 (42.3, 45.0)	33.3 (32.0, 34.6)	14.9 (13.9, 15.9)	8.3 (7.6, 9.1)
2008, n = 6116	41.8 (40.4, 43.2)	33.7 (32.4, 35.0)	15.8 (14.8,16.9)	8.7 (7.9, 9.5)

Values are weighted percentages, with 95% confidence intervals in parentheses, derived by using the HRS respondent population weights of each year to adjust for the sampling design of survey.

* P value < 0.001, derived from the Wald chi-square tests which were performed to examine group differences in proportions of characteristics across years of survey. P-values refer to whether the proportions or characteristics were significantly different among groups between years.

† P value = 0.01.

### Disability Trends

Among ADL and IADL disabilities, with the exception of money management, the prevalence of the various disabilities examined during the study time period remained stable (Table 1). The proportion of respondents reporting any ADL disability did not change consistently over the study period, from 19.2% in 1998 to 17.4% in 2004, to 18.4% in 2008 (Table 4 and Figure 1C in the online supplement; p = 0.01), and there was a non-significant decline in the proportion reporting any IADL disability, from 17.8% in 1998 to 17.7% in 2004 to 16.6% in 2008 (p = 0.06). The proportion of respondents reporting any ADL or IADL disability declined initially from 26.3% in 1998 to 25.3% in 2004, but remained steady as of 2008 at 25.4% (p = 0.23).

**Table 4:** Percentage of respondents with disability in activities of daily living (ADL), instrumental activities of daily living (IADL) and either ADL or IADL, stratified by age and by sex

	ADL or IADL Disability, % (95% Confidence Interval)		
	**Any ADL**	**Any IADL**	**Either ADL or IADL**

**Overall**			
1998, n = 10390	19.2 (18.4, 20.0)	17.8 (17.0, 18.6)	26.3 (25.4, 27.2)

2004, n = 10621	17.4 (16.6, 18.2)	17.7 (16.9, 18.5)	25.3 (24.4, 26.2)
2008, n = 10557	18.4 (17.6, 19.2)	16.6 (15.8, 17.4)	25.4 (24.5, 26.3)

	p = 0.01	p = 0.06	p = 0.25

**Respondent Age**			

**65-69 years**			

1998, n = 3018	12.4 (11.2, 13.7)	9.1 (8.0, 10.2)	16.3 (14.9, 17.8)
2004, n = 3392	11.3 (10.2, 12.6)	9.2 (8.2, 10.4)	15.5 (14.2, 16.9)
2008, n = 3128	13.1 (11.7, 14.7)	9.5 (8.3, 10.8)	17.0 (15.5, 18.7)

	p = 0.15	p = 0.89	p = 0.31

**70-74 years**			

1998, n = 2720	15.3 (13.9, 16.8)	12.6 (11.0, 13.5)	20.4 (18.8, 22.0)
2004, n = 2656 2008, n = 2908	13.4 (12.0, 14.9) 13.2 (11.9, 14.6)	12.2 (10.9, 13.6) 11.7 (10.5, 13.1)	19 (17.4, 20.7) 19 (17.4, 20.6)

	p = 0.08	p = 0.84	P = 0.38

**75-79 years**			

1998, n = 2284	19.4 (17.7, 21.3)	19.1 (17.4, 20.9)	27.6 (25.6, 29.6)
2004, n = 2031	17.1 (15.3, 18.9)	17.6 (15.8, 19.4)	25.7 (23.7, 27.8)
2008, n = 2180	19.2 (17.4, 21.2)	15.1 (13.4, 16.8)	25.4 (23.4, 27.5)

	p = 0.12	p = 0.01	p = 0.27

**80-84 years**			

1998, n = 1465	26.5 (24.1, 29.1)	26.7 (24.2, 29.2)	37.6 (34.9, 40.4)
2004, n = 1609	22.2 (20.1, 24.5)	23.4 (21.1, 25.8)	32.8 (30.3, 35.4)
2008, n = 1517	23.1 (22.4, 25.2)	24.6 (22.3, 27.1)	35.1 (32.5, 37.8)

	p = 0.03	p = 0.17	p = 0.05

**≥ 85 years**			

1998, n = 1306	41 (38.0, 44.2)	46.6 (43.3, 49.6)	56.4 (53.2, 59.5)
2004, n = 1425	36.6 (33.7, 39.3)	44.7 (41.5, 47.7)	54.0 (50.9, 57.1)
2008, n = 1588	36.4 (35.9, 39.4)	39.1 (41.0, 44.5)	50.2 (47.2, 53.1)

	p = 0.08	p = 0.002	p = 0.02

**Respondent Sex**			

**Male**			

1998, n = 4463	15.6 (14.5, 16.8)	15.3 (14.2, 16.5)	23.0 (21.7, 24.3)
2004, n = 4595	14.9 (13.9, 16.1)	15.9 (14.7, 17.0)	23.2 (21.9, 24.5)
2008, n = 4441	15.0 (13.9, 16.2)	13.8 (12.7, 14.9)	21.6 (20.3, 23.0)

	p = 0.65	p = 0.02	p = 0.18

**Female**			

1998, n = 5927	21.7 (20.6, 22.9)	19.6 (18.6, 20.7)	28.7 (27.5, 30.0)
2004, n = 6026	19.3 (18.3, 20.4)	19.1 (18.0, 20.2)	26.9 (25.7, 28.2)
2008, n = 6116	21.0 (19.8, 22.1)	18.8 (17.7, 19.9)	28.3 (27.0, 29.5)

	p = 0.01	p = 0.53	p = 0.11

Values are weighted percentages, with 95% confidence intervals in parentheses, derived by using the HRS respondent population weights of each year to adjust for the sampling design of survey.

Note: P values were derived from the Wald chi-square tests which were performed to examine group differences in proportions of characteristics across years of survey. P-values refer to whether the proportions of characteristics were significantly different among groups between years.

### Age-Stratified Trends

For chronic disease trends, there was an increasing trend towards higher proportion of respondents with higher number of chronic diseases among all age groups (Table 2). For impairment trends, there were no consistent patterns observed among respondents in all age group except among those aged 65-69, where the proportion with no impairment declined from 58.8% in 1998 to 53.0% in 2008 and the proportion of respondents with 1 and 2 impairments increased (Table 3). For disability trends, there was a significant decline in IADL disability (p < 0.01) and any disability (p = 0.02) among the oldest age group (85 and above), as well as in IADL disability among those aged 75-79 (p < 0.01; Table 4).

### Sex-Stratified Trends

For chronic disease trends, the increase in multiple chronic disease prevalence was similar among men and women (Table 2). For impairments, there was a trend towards increasing multiple impairments among women, but not men (Table 3). For disability trends, among both men and women, there were fluctuations in the prevalence of disability but no consistent trends were observed (Table 4).

### Sensitivity Analysis

Removing each individual chronic disease in the count for multiple chronic diseases demonstrated that no individual chronic disease was responsible for the observed trends. For impairment trends, the significant increases in multiple impairments appear to be largely due to the rise in the prevalence in urinary incontinence. Other individual impairments did not account for any significant trends in multiple impairments.

## Discussion

Our analysis of recent concurrent trends in chronic disease, impairment and disability demonstrate that the prevalence of chronic diseases and multiple chronic diseases has increased over time among older adults. In contrast, the prevalence of impairments and multiple impairments, except urinary incontinence, have largely remained stable, as have the prevalence of ADL and IADL disabilities. Overall, despite the increase in the prevalence of chronic disease and multiple chronic diseases, we did not observe a concurrent increase in the prevalence of impairment or disability. Our findings are consistent with older trends, demonstrating the rising prevalence of chronic disease among older adults, as well the relative stability of impairment trends and the declining trends in the prevalence of disability, but clarify that these trends remain consistent when examined concurrently and have persisted as of 2008.

We also found that there were age and gender disparities in disability trends. Disability for IADL and ADL has declined mostly among the oldest old, and the prevalence of disability was higher in women than men at every time point. For impairments, we observed increases in impairments among the 65-69 age group and among women, which we found were due to the increase in the prevalence of urinary incontinence. Although the overall prevalence of multiple impairments did not increase over time, we observed that the prevalence of multiple impairments remained high particularly among the oldest old group.

Our finding that the prevalence of impairments and disability has largely not been increasing despite growing burden of chronic disease is consistent with prior work on trends. Prior reports have found an increase in chronic disease prevalence throughout the 1990s and 2000s [4,9]. Our findings add to this literature not only by examining more recent data, but also by using a single population to also estimate impairment and disability prevalence. That impairment trends were largely stable while chronic disease prevalence increased suggests that chronic diseases were becoming less likely to be associated with impairments over time. Finally, our finding that disability trends were similarly stable is consistent with prior reports which showed that disability has slightly declined over the years, but has been stable more recently [10,11,14].

There are several possible explanations for our finding that impairment and disability patterns over time have not changed significantly despite the dramatic increase in chronic disease prevalence among older adults, particularly given the relationship between chronic diseases and impairments. First, increased screening of chronic disease is likely to identify more disease, but of a less severe variety, such that adults with chronic disease now appear to live with the disease for longer periods of time before causing impairment. Second, either separate from or perhaps partly due to chronic diseases being diagnosed at an earlier stage, chronic diseases may be better managed, reducing the risk of developing impairment from these chronic diseases. For instance, the availability of greater numbers of pharmaceutical treatments with differing mechanisms of action for coronary heart disease, hypertension, diabetes, COPD and many other diseases over the past 20 years may have improved the management of these diseases among older adults. Examples of diseases which might respond to treatment include arthritis treatment improving mobility; improved treatment of diabetes (better control) may lead to improvement in eye sight, and may also be related to cognition and incontinence. Improved management of chronic lung disease may also improve mobility. Third, improved management of chronic disease and screening for potential sequelae of chronic disease may have reduced the likelihood or impact of impairment arising from disease. An example of this would be improved podiatric care and retinopathy screening among adults with diabetes. Higher education levels over the recent past may also have contributed to greater patient self-seeking of preventive care and improved self-management of chronic disease.

The rise in prevalence in multiple chronic disease among older adults in the U.S. is likely to be associated with rising health care utilization and costs, although thus far, has not been associated with increased rates of impairments and disability. This may seem to suggest that our health care system is working effectively. However, when we consider how the U.S. population is rapidly aging [37], these rates may be misleading. The absolute number of older adults with multiple chronic diseases, impairment and disability all continue to climb even though prevalence rates may be stable for impairments and disability. These trends have substantial ramifications on overall costs incurred by the older adult population. Therefore, it is important to continue to find ways, such as new models of care or reimbursement structures, to effectively manage this expanding population with multiple chronic diseases with substantial burden of disability.

Our study has several strengths. First, we used a large nationally representative database which contains data on chronic diseases, impairments, and disability, allowing for the most up-to-date concurrent examination of national trends to enhance our understanding of health problems faced by older adults today. Due to the aging of the sample in the cohort and replenishment of new birth cohorts, we were able to compare rates of chronic disease, impairments, and disability when the sample was stratified by age and sex. However, our study has limitations as well. First, in order to utilize sampling weights to estimate nationally-representative prevalence rates for all 3 survey waves, we limited our study to community-dwelling respondents and excluded respondents residing in nursing homes. Because the overall size of the nursing home population in the United States has declined over the study period [38], we would expect that adults previously cared for in nursing homes would be more likely to be in the community and thus that prevalence rates of impairment or disability would have risen, rather than remained flat as we observed. Second, our measures of chronic disease, impairment, and disability were based on self-report of conditions. The HRS provides unique longitudinal survey data to identify population health trends. In population-based cohorts, self-reporting of health conditions is an accepted methodology for large, nationally representative survey for which detailed chart review is not feasible and the concordance between self report and medical record review is generally good (κ = 0.60) [39]. Although the survey is limited by its use of self report to ascertain chronic disease, impairment and disability, prior studies have suggested that self-report provides accurate prevalence estimates for all three [39-44].

## Conclusions

A main goal of successful aging is the maintenance of independence [45]. In fact, a main aspect of the health care of older adults is the attention paid to maintain functional ability; preventing disability by the effective management of chronic diseases and impairments is of paramount importance to the care of older adults so that they can continue to live independently in the community for as long as possible. Our study shows that multiple chronic diseases have become increasingly prevalent and impairments and disability continue to be a substantial, although not rising, burden among older adults. The aging, U.S. population continues to need high-quality care, focused on managing multiple chronic diseases and preventing impairment and disability.

## Abbreviations

(ADL): Activities of daily living; (BMI): body mass index; (CI): confidence interval; (COPD): chronic obstructive pulmonary disease; (HRS): Health and Retirement Study; (IADL): instrumental activities of daily living.

## Competing interests

The authors declare that they have no competing interests.

## Authors' contributions

Specific contributions by individual authors are described below:

*Study Concept and design: *WH, JR, AS; *Acquisition of Data: *WH; *Analysis and interpretation of data: *WH, JR, KB, AS; *Drafting of the manuscript: *WH, JR; *Critical revision of the manuscript for important intellectual content: *WH, JR, KB, AS; *Statistical Analysis: *WH; *Study Supervision: *JR, AS. All authors read and approved the final manuscript.

## Pre-publication history

The pre-publication history for this paper can be accessed here:

http://www.biomedcentral.com/1471-2318/11/47/prepub
